# Supplementary material for: Target Selection for T-Cell Therapy in Epithelial Ovarian Cancer: Systematic Prioritization of Self-Antigens
Source: Int J Mol Sci. 2023 Jan 24;24(3):2292. doi: 10.3390/ijms24032292 (PMC9916968; doi:10.3390/ijms24032292)
Supplement: Supplementary file 1 [file ijms-24-02292-s001.zip › Supplemental Figures Kaplan-Meier Plots.pptx]

## Slide 1
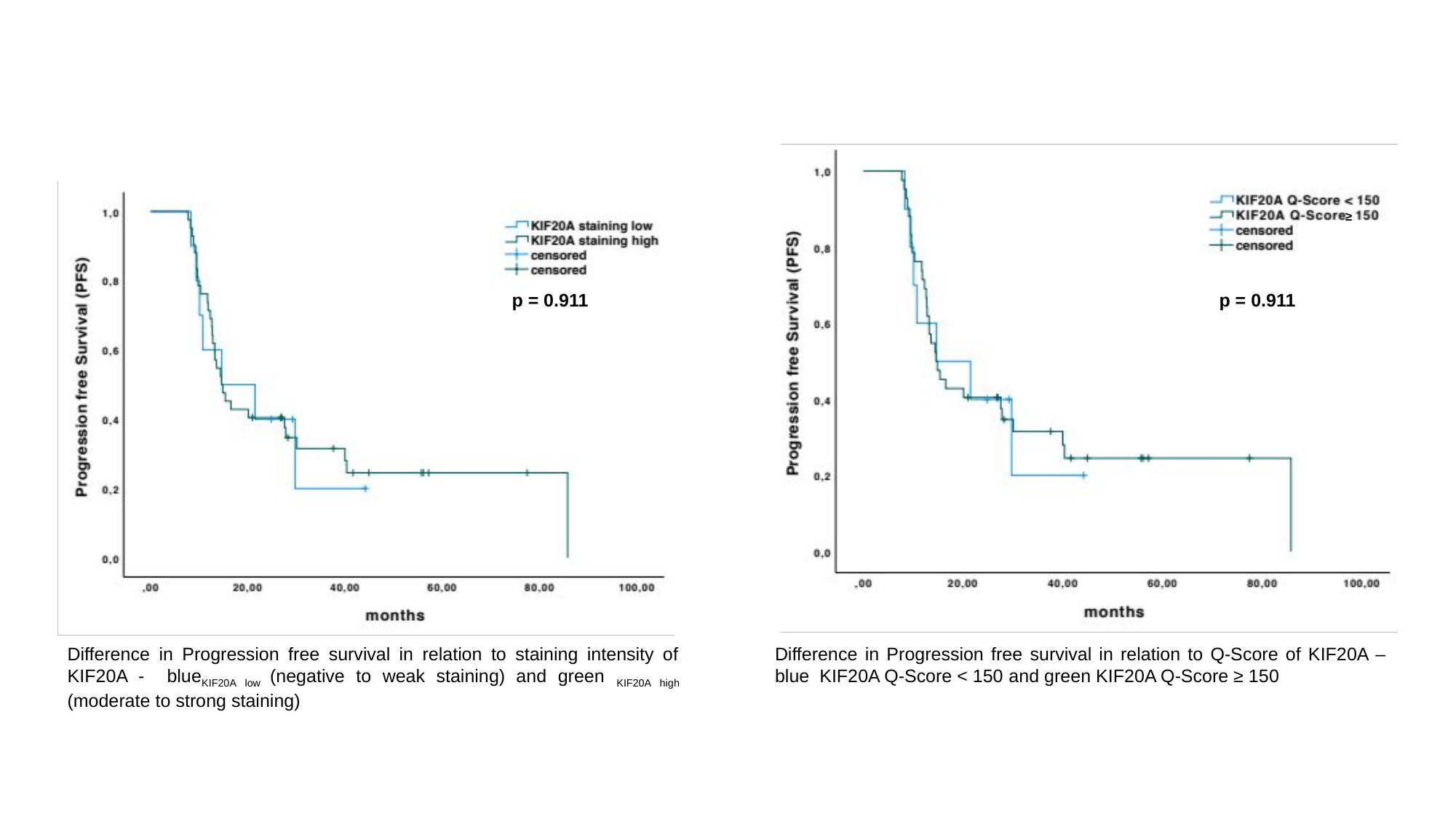

≥
p = 0.911
p = 0.911
Difference in Progression free survival in relation to Q-Score of KIF20A – blue KIF20A Q-Score < 150 and green KIF20A Q-Score ≥ 150
Difference in Progression free survival in relation to staining intensity of KIF20A - blueKIF20A low (negative to weak staining) and green KIF20A high (moderate to strong staining)

## Slide 2
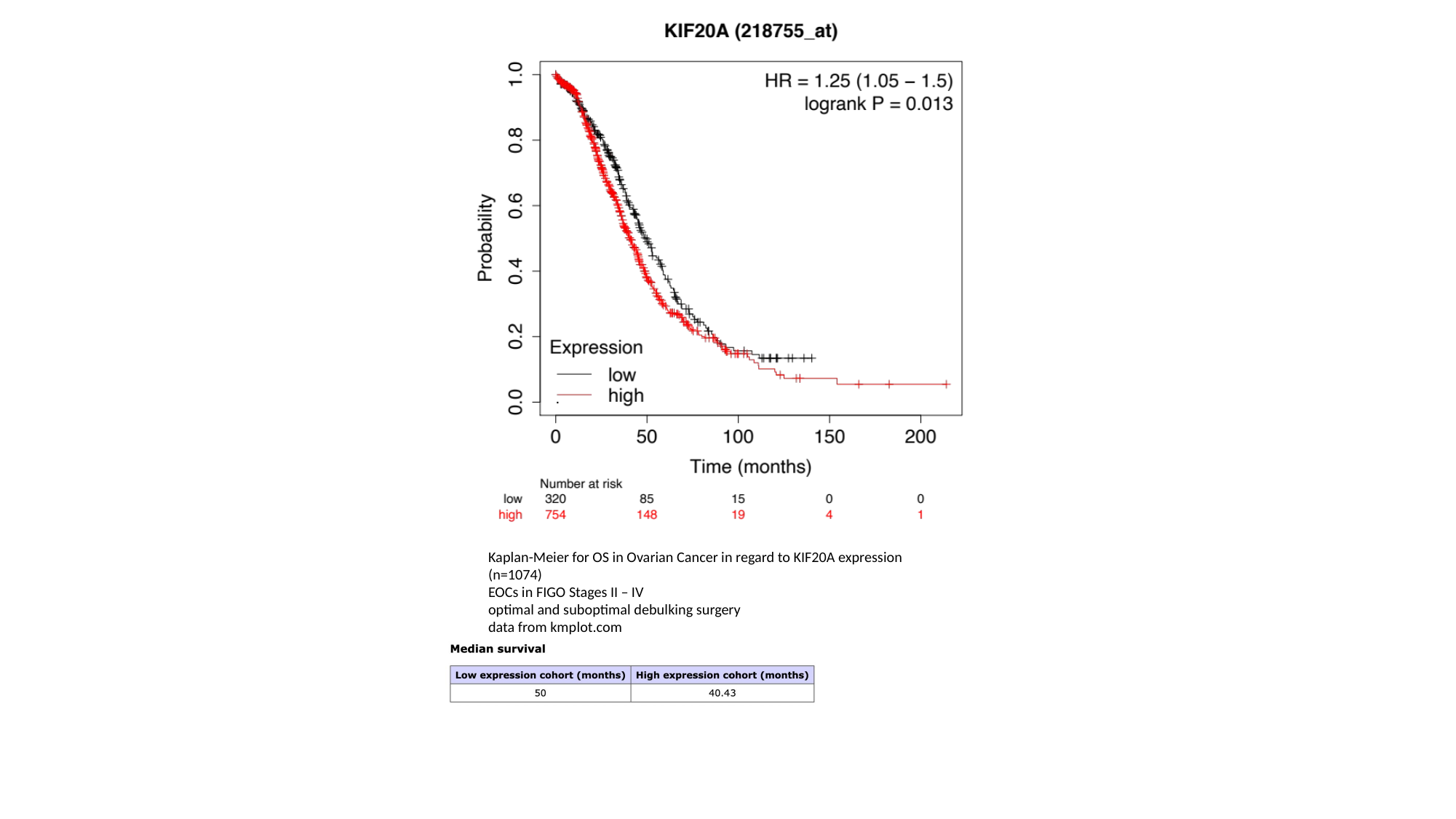

Kaplan-Meier for OS in Ovarian Cancer in regard to KIF20A expression (n=1074)
EOCs in FIGO Stages II – IV
optimal and suboptimal debulking surgery
data from kmplot.com

## Slide 3
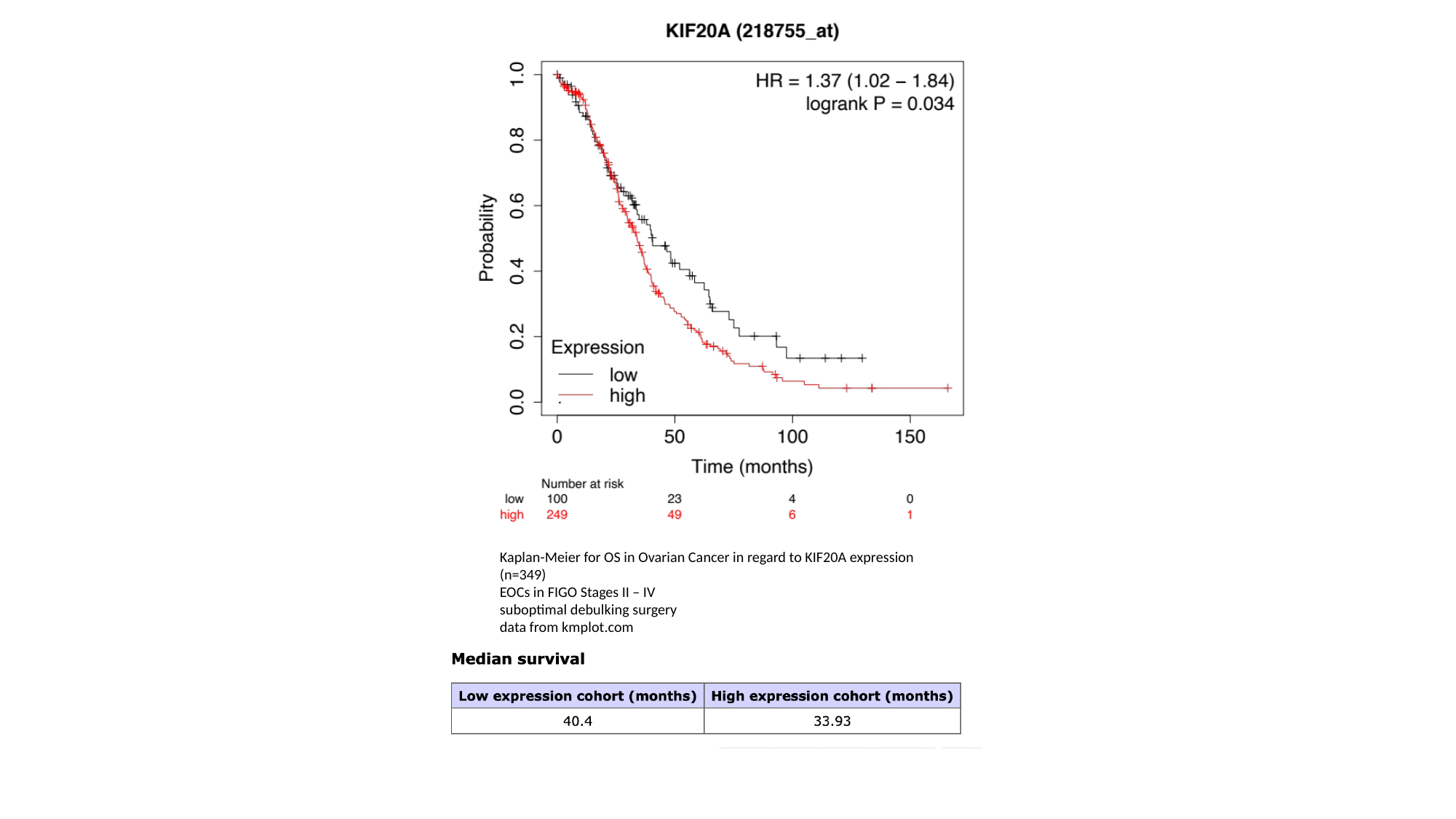

Kaplan-Meier for OS in Ovarian Cancer in regard to KIF20A expression (n=349)
EOCs in FIGO Stages II – IV
suboptimal debulking surgery
data from kmplot.com

## Slide 4
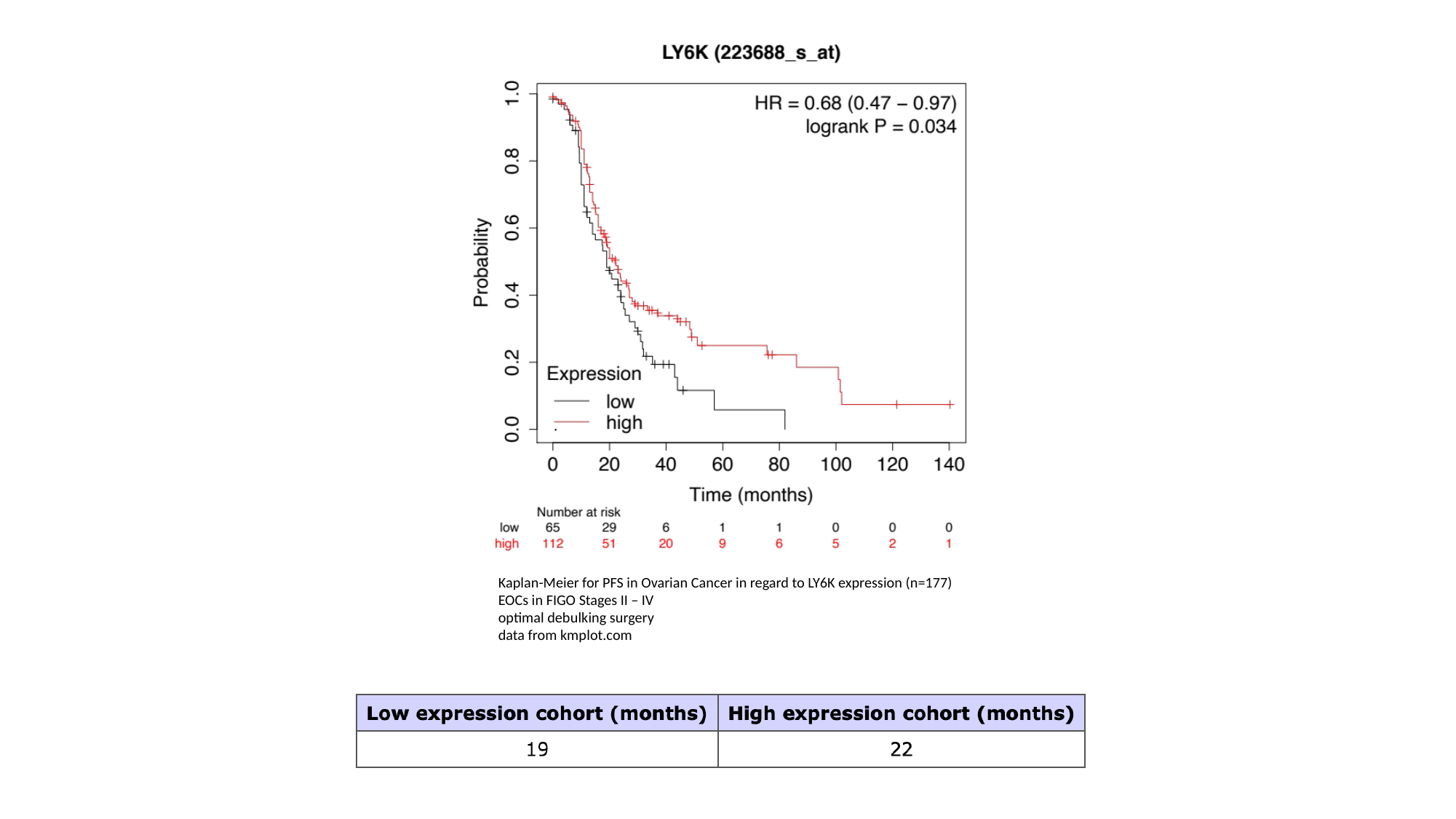

Kaplan-Meier for PFS in Ovarian Cancer in regard to LY6K expression (n=177)
EOCs in FIGO Stages II – IV
optimal debulking surgery
data from kmplot.com
