# Supplementary material for: Target Selection for T-Cell Therapy in Epithelial Ovarian Cancer: Systematic Prioritization of Self-Antigens
Source: Int J Mol Sci. 2023 Jan 24;24(3):2292. doi: 10.3390/ijms24032292 (PMC9916968; doi:10.3390/ijms24032292)
Supplement: Supplementary file 1 [file ijms-24-02292-s001.zip › Supplemental Table S2 - TAA references .pdf]

Supplemental Table S2 TAA-references

(-) group Antigen	No. of references	Author	(+) group Antigen	No. of references	Author
**PRAME**	**6**	Al-Khadairi, Naik (1), Kloudova, Hromadkova (2), Kreuzinger, von der Decken (3), Orlando, Miele (4), Xu, Zou (5), Zhang, Barger (6)	**MSLN**	**4**	Anderson, Voillet (7), Chang, Chen (8), Hassan, Thomas (9), Hilliard (10)
**MUC16**	**4**	Aithal, Rauth (11), Felder, Kapur (12), Rao, Fernandez-Tejada (13)	**MUC1**	**3**	Kloudova, Hromadkova (2), Deng, Wang (14), Dobrzanski, Rewers-Felkins (15)
**CCNA1**	**4**	Huang, Yang (16), Arsenic, Braicu (17), Ochsenreither, Majeti (18) Teck, Urban (19)	**MMP7**	**5**	Al-Alem, McCord (20), Guo, Zhang (21), Sillanpaa, Anttila (22), Wang, So (23), Yokoyama, Grunebach (24)
**KIF20A**	**5**	Imai, Hirata (25), Kawai, Shibata (26), Li, Zhang (27), Schiewek, Schumacher (28), Shi, Huang (29)	**WT1**	**3**	Kloudova, Hromadkova (2), Taube, Denkert (30), Lu, Gu (31)
**IDO1**	**6**	Inaba, Ino (32), Sorensen, Berge-Hansen (33), Bahrami, Hosseini (34), Kristeleit, Davidenko (35), Schuster, Peper (36), Stapelberg, Zobalova (37)	**SOX4**	**2**	Liu, Wang (38), Sun and Fan (39)
**DCD2**	**1**	Van Den Eynde, Gaugler (40)	**CCNB1**	**2**	Kao, Marto (41), Zheng, Hu (42)
**GPR143**	**2**	Touloukian, Leitner (43) Bai, Xie (44)	**TOP2A**	**3**	Gao, Zhao (45), Jain, Zhang (46), Park, Kim (47)
**MAGEA4**	**7**	Bhan, Chuang (48), Daudi, Eng (49), Garcia-Soto, Schreiber (50), Kageyama, Ikeda (51), Sanderson, Crowley (52), Yakirevich, Sabo (53), Zimmermann, Imig (54)	**BIRC5**	**7**	Kloudova, Hromadkova (2), Berinstein, Karkada (55), Gil-Kulik, Krzyzanowski (56), He, Yang (57), Plewka, Jakubiec-Bartnik (58), Yamamoto, Suzuki (59), Zhao, Wang (60)
**SPA17**	**6**	Brunette, Mhawech-Fauceglia (61), Chiriva-Internati, Weidanz (62), Gao, Xiang (63), Kanduc (64), Straughn, Shaw (65), Vermeij, Daemen (66)	**LRPAP1**	**1**	Marijt, Blijleven (67)
**CTAG2**	**3**	Odunsi, Jungbluth (68), McCormack, Adams (69), Rapoport, Stadtmauer (70)	**GPC3**	**4**	Cheng, Huang (71), Liu, Zheng (72), Pantanowitz and Otis (73), Shimizu, Suzuki (74)
**MAGEC2**	**5**	Godelaine, Carrasco (75), Lamers, van Steenbergen-Langeveld (76), Ma, Germeau (77), Song, Hao (78), Straetemans, van Brakel (79)	**ENAH**	**1**	Di Modugno, Caprara (80)
**SOX11**	**6**	Brennan, Ek (81), Choi, Seong (82), Davidson, Holth (83), Fang, Liu (84), Schmitz, Wehner (85), Sernbo, Gustavsson (86)	**STEAP1**	**4**	Hayashi, Kumai (87), Jiao, Huang (88), Moreaux, Kassambara (89), Vaghjiani, Talma (90)
**CT45**	**4**	Chen, Hsu (91), Coscia, Lengyel (92), Shang, Gao (93), Zhang, Barger (94)	**ETV5**	**1**	Llaurado, Abal (95)

(-) group Antigen	No. of references	Author and year	(+) group Antigen	No. of references	Author and year
**IMP3**	**6**	Chiste, Alexis (96), El-Balat, Sanger (97), Hsu, Shen (98), Kobel, Xu (99), Lu, Wang (100), Tomita, Harao (101)	**MGAT5**	**0**	/
**MAGEC1**	**3**	Daudi, Eng (49), Zimmermann, Imig (54), Khalaf, Garg (102)	**NECTIN4**	**5**	Bekos, Muqaku (103), Boylan, Buchanan (104), Derycke, Pambuccian (105), Lopez, Ghidouche (106), Sethy, Goutam (107)
**LY6K**	**5**	AlHossiny, Luo (108), Benti, Tiwari (109), Luo, McGarvey (110), Sastry, Wan (111), Tomita, Yuno (112)	**ADAM17**	**1**	Sinnathamby (2010) Sinnathamby, Zerfass (113)
**HLA-G**	**0**	/	**EZH2**	**0**	/
**MAGEA1**	**4**	Daudi, Eng (49), Gillespie, Rodgers (114), Kerkar, Wang (115), Zhang, Zhou (116)			
**KAAG1**	**0**	/			
**OCA2**	**0**	/			
**CCDC110**	**1**	Monji (2004) Monji, Nakatsura (117)			

Type of Paper [1-117]

**Expression** (41)	
IHC (31)	Arsenic (2015), Li (2018), Kawai (2018), Inaba (2017), Daudi (2014), Garcia-Soto (2017), Yakirevich (2004), Zimmermann (2013), Brunette (2018), Vermeij (2010), Kumara (2012), Sernbo (2011), Brennan (2009), Davidson (2015), Zhang (2016), Chen (2009), Lu (2018), Köbel (2009), Chisté (2014), El-Balat (2017), Zhang (2010), Gillespie (1998), Kerkar (2016), Silanpäa (2006), Taube (2016), Zheng (2009), Plewka (2015), Pantanowitz (2015), Jiao (2020), Bekos (2019), DeRycke (2010),
RNA seq. (9)	Zhang (2016), Kloudova (2016), Huang (2016), Schiewek (2018), Bahrami (2017), Stapelberg (2013), Straughn Jr. (2004), Luo (2016), Moreaux (2012)
**T-Cells/TCR** (31)	Kreuzinger (2019), Orlando (2018), Ochsenreither (2012), Imai (2011), Sörensen (2009), van den Eynde (1999), Touloukian (2003), Sanderson (2020), Chiriva-Internati (2008), Kanduc (2019), McCormack (2012), Rapoport (2015), Straetemans (2012), Lamers (2014), Godelaine (2006), Wen (2011), Ma (2004), Schmitz (2006), Coscia (2018), Tomita (2011), Khalaf (2019), Tomita (2014), Monji (2004), Anderson (2019), Yokoyama (2008), Kao (2001), Ramakrishna (2003), Marijt (2018), Hayashi (2011), Lopez (2016), Sinnathamby (2010)
**Immunotherapy** (6)	Kristeleit (2017), Kageyama (2015), Hassan (2016), Dobrzanski (2010), Beirnstein (2015), Shimizu (2019),
**Oncogenicity** (33)	Rao (2020), Aithal (2018), Shi (2016), Bai (2014), Bhan (2012), Gao (2018), Song (2017), Fang (2017), Choi (2014), Shang (2014), Hsu (2015), Benti (2020) Sastry (2020), Al-Hossiny (2016), Chang (2012), Hilliard (2018), Deng (2013), Guo (2020), Wang (2004), Al-Alem (2013), Sun (2019), Liu (2018), Zhao (2019), Gil-Kulik (2018), Yamamoto (2018), Chen (2017), Liu (2015), DiModungo (2018), Vaghijani (2009), LLauradó (2011), Boylan (2017), Sethy (2019), Jain (2013)
**Review** (6)	Al-Khadiri (2019), Xu (2020), Felder (2014), Odunsi (2003), Lu (2018), He (2018),

1. Al-Khadairi G, Naik A, Thomas R, Al-Sulaiti B, Rizly S, Decock J. PRAME promotes epithelial-to-mesenchymal transition in triple negative breast cancer. J Transl Med. 2019;17(1):9.
2. Kloudova K, Hromadkova H, Partlova S, Brtnicky T, Rob L, Bartunkova J, et al. Expression of tumor antigens on primary ovarian cancer cells compared to established ovarian cancer cell lines. Oncotarget. 2016;7(29):46120-6.
3. Kreuzinger C, von der Decken I, Wolf A, Gamperl M, Koller J, Karacs J, et al. Patient-derived cell line models revealed therapeutic targets and molecular mechanisms underlying disease progression of high grade serous ovarian cancer. Cancer Lett. 2019;459:1-12.
4. Orlando D, Miele E, De Angelis B, Guercio M, Boffa I, Sinibaldi M, et al. Adoptive Immunotherapy Using PRAME-Specific T Cells in Medulloblastoma. Cancer Res. 2018;78(12):3337-49.

5. Xu Y, Zou R, Wang J, Wang ZW, Zhu X. The role of the cancer testis antigen PRAME in tumorigenesis and immunotherapy in human cancer. Cell Prolif. 2020;53(3):e12770.
6. Zhang W, Barger CJ, Eng KH, Klinkebiel D, Link PA, Omilian A, et al. PRAME expression and promoter hypomethylation in epithelial ovarian cancer. Oncotarget. 2016;7(29):45352-69.
7. Anderson KG, Voillet V, Bates BM, Chiu EY, Burnett MG, Garcia NM, et al. Engineered Adoptive T-cell Therapy Prolongs Survival in a Preclinical Model of Advanced-Stage Ovarian Cancer. Cancer Immunol Res. 2019;7(9):1412-25.
8. Chang MC, Chen CA, Chen PJ, Chiang YC, Chen YL, Mao TL, et al. Mesothelin enhances invasion of ovarian cancer by inducing MMP-7 through MAPK/ERK and JNK pathways. Biochem J. 2012;442(2):293-302.
9. Hassan R, Thomas A, Alewine C, Le DT, Jaffee EM, Pastan I. Mesothelin Immunotherapy for Cancer: Ready for Prime Time? J Clin Oncol. 2016;34(34):4171-9.
10. Hilliard TS. The Impact of Mesothelin in the Ovarian Cancer Tumor Microenvironment. Cancers (Basel). 2018;10(9).
11. Aithal A, Rauth S, Kshirsagar P, Shah A, Lakshmanan I, Junker WM, et al. MUC16 as a novel target for cancer therapy. Expert Opin Ther Targets. 2018;22(8):675-86.
12. Felder M, Kapur A, Gonzalez-Bosquet J, Horibata S, Heintz J, Albrecht R, et al. MUC16 (CA125): tumor biomarker to cancer therapy, a work in progress. Mol Cancer. 2014;13:129.
13. Rao TD, Fernandez-Tejada A, Axelrod A, Rosales N, Yan X, Thapi S, et al. Antibodies Against Specific MUC16 Glycosylation Sites Inhibit Ovarian Cancer Growth. ACS Chem Biol. 2017;12(8):2085-96.
14. Deng J, Wang L, Chen H, Li L, Ma Y, Ni J, et al. The role of tumour-associated MUC1 in epithelial ovarian cancer metastasis and progression. Cancer Metastasis Rev. 2013;32(3-4):535-51.
15. Dobrzanski MJ, Rewers-Felkins KA, Quinlin IS, Samad KA, Phillips CA, Robinson W, et al. Autologous MUC1-specific Th1 effector cell immunotherapy induces differential levels of systemic TReg cell subpopulations that result in increased ovarian cancer patient survival. Clin Immunol. 2009;133(3):333-52.
16. Huang KC, Yang J, Ng MC, Ng SK, Welch WR, Muto MG, et al. Cyclin A1 expression and paclitaxel resistance in human ovarian cancer cells. Eur J Cancer. 2016;67:152-63.
17. Arsenic R, Braicu EI, Letsch A, Dietel M, Sehouli J, Keilholz U, et al. Cancer-testis antigen cyclin A1 is broadly expressed in ovarian cancer and is associated with prolonged time to tumor progression after platinum-based therapy. BMC Cancer. 2015;15:784.
18. Ochsenreither S, Majeti R, Schmitt T, Stirewalt D, Keilholz U, Loeb KR, et al. Cyclin-A1 represents a new immunogenic targetable antigen expressed in acute myeloid leukemia stem cells with characteristics of a cancer-testis antigen. Blood. 2012;119(23):5492-501.
19. Teck AT, Urban S, Quass P, Nelde A, Schuster H, Letsch A, et al. Cancer testis antigen Cyclin A1 harbors several HLA-A*02:01-restricted T cell epitopes, which are presented and recognized in vivo. Cancer Immunol Immunother. 2020;69(7):1217-27.
20. Al-Alem LF, McCord LA, Southard RC, Kilgore MW, Curry TE, Jr. Activation of the PKC pathway stimulates ovarian cancer cell proliferation, migration, and expression of MMP7 and MMP10. Biol Reprod. 2013;89(3):73.
21. Guo F, Zhang K, Li M, Cui L, Liu G, Yan Y, et al. miR5083p suppresses the development of ovarian carcinoma by targeting CCNA2 and MMP7. Int J Oncol. 2020;57(1):264-76.
22. Sillanpaa S, Anttila M, Voutilainen K, Ropponen K, Turpeenniemi-Hujanen T, Puistola U, et al. Prognostic significance of matrix metalloproteinase-9 (MMP-9) in epithelial ovarian cancer. Gynecol Oncol. 2007;104(2):296-303.
23. Wang FQ, So J, Reierstad S, Fishman DA. Matrilysin (MMP-7) promotes invasion of ovarian cancer cells by activation of progelatinase. Int J Cancer. 2005;114(1):19-31.
24. Yokoyama Y, Grunebach F, Schmidt SM, Heine A, Hantschel M, Stevanovic S, et al. Matrilysin (MMP-7) is a novel broadly expressed tumor antigen recognized by antigen-specific T cells. Clin Cancer Res. 2008;14(17):5503-11.
25. Imai K, Hirata S, Irie A, Senju S, Ikuta Y, Yokomine K, et al. Identification of HLA-A2-restricted CTL epitopes of a novel tumour-associated antigen, KIF20A, overexpressed in pancreatic cancer. Br J Cancer. 2011;104(2):300-7.
26. Kawai Y, Shibata K, Sakata J, Suzuki S, Utsumi F, Niimi K, et al. KIF20A expression as a prognostic indicator and its possible involvement in the proliferation of ovarian clearcell carcinoma cells. Oncol Rep. 2018;40(1):195-205.
27. Li H, Zhang W, Sun X, Chen J, Li Y, Niu C, et al. Overexpression of kinesin family member 20A is associated with unfavorable clinical outcome and tumor progression in epithelial ovarian cancer. Cancer Manag Res. 2018;10:3433-50.
28. Schiewek J, Schumacher U, Lange T, Joosse SA, Wikman H, Pantel K, et al. Clinical relevance of cytoskeleton associated proteins for ovarian cancer. J Cancer Res Clin Oncol. 2018;144(11):2195-205.
29. Shi C, Huang D, Lu N, Chen D, Zhang M, Yan Y, et al. Aberrantly activated Gli2-KIF20A axis is crucial for growth of hepatocellular carcinoma and predicts poor prognosis. Oncotarget. 2016;7(18):26206-19.
30. Taube ET, Denkert C, Sehouli J, Kunze CA, Dietel M, Braicu I, et al. Wilms tumor protein 1 (WT1)-- not only a diagnostic but also a prognostic marker in high-grade serous ovarian carcinoma. Gynecol Oncol. 2016;140(3):494-502.
31. Lu J, Gu Y, Li Q, Zhong H, Wang X, Zheng Z, et al. Wilms' tumor 1 (WT1) as a prognosis factor in gynecological cancers: A meta-analysis. Medicine (Baltimore). 2018;97(28):e11485.
32. Inaba T, Ino K, Kajiyama H, Yamamoto E, Shibata K, Nawa A, et al. Role of the immunosuppressive enzyme indoleamine 2,3-dioxygenase in the progression of ovarian carcinoma. Gynecol Oncol. 2009;115(2):185-92.
33. Sorensen RB, Berge-Hansen L, Junker N, Hansen CA, Hadrup SR, Schumacher TN, et al. The immune system strikes back: cellular immune responses against indoleamine 2,3-dioxygenase. PLoS One. 2009;4(9):e6910.
34. Bahrami B, Hosseini A, Talei AR, Ghaderi A, Razmkhah M. Adipose Derived Stem Cells Exert Immunomodulatory Effects on Natural Killer Cells in Breast Cancer. Cell J. 2017;19(1):137-45.

35. Kristeleit R, Davidenko I, Shirinkin V, El-Khouly F, Bondarenko I, Goodheart MJ, et al. A randomised, open-label, phase 2 study of the IDO1 inhibitor epacadostat (INCB024360) versus tamoxifen as therapy for biochemically recurrent (CA-125 relapse)-only epithelial ovarian cancer, primary peritoneal carcinoma, or fallopian tube cancer. Gynecol Oncol. 2017;146(3):484-90.
36. Schuster H, Peper JK, Bosmuller HC, Rohle K, Backert L, Bilich T, et al. The immunopeptidomic landscape of ovarian carcinomas. Proc Natl Acad Sci U S A. 2017;114(46):E9942-E51.
37. Stapelberg M, Zobalova R, Nguyen MN, Walker T, Stantic M, Goodwin J, et al. Indoleamine-2,3-dioxygenase elevated in tumor-initiating cells is suppressed by mitocans. Free Radic Biol Med. 2014;67:41-50.
38. Liu Y, Wang Y, Yao D, Cui D. LncSOX4 serves an oncogenic role in the tumorigenesis of epithelial ovarian cancer by promoting cell proliferation and inhibiting apoptosis. Mol Med Rep. 2018;17(6):8282-8.
39. Sun D, Fan XH. LncRNA SNHG12 accelerates the progression of ovarian cancer via absorbing miRNA-129 to upregulate SOX4. Eur Rev Med Pharmacol Sci. 2019;23(6):2345-52.
40. Van Den Eynde BJ, Gaugler B, Probst-Kepper M, Michaux L, Devuyst O, Lorge F, et al. A new antigen recognized by cytolytic T lymphocytes on a human kidney tumor results from reverse strand transcription. J Exp Med. 1999;190(12):1793-800.
41. Kao H, Marto JA, Hoffmann TK, Shabanowitz J, Finkelstein SD, Whiteside TL, et al. Identification of cyclin B1 as a shared human epithelial tumor-associated antigen recognized by T cells. J Exp Med. 2001;194(9):1313-23.
42. Zheng H, Hu W, Deavers MT, Shen DY, Fu S, Li YF, et al. Nuclear cyclin B1 is overexpressed in low-malignant-potential ovarian tumors but not in epithelial ovarian cancer. Am J Obstet Gynecol. 2009;201(4):367 e1-6.
43. Touloukian CE, Leitner WW, Schnur RE, Robbins PF, Li Y, Southwood S, et al. Normal tissue depresses while tumor tissue enhances human T cell responses in vivo to a novel self/tumor melanoma antigen, OA1. J Immunol. 2003;170(3):1579-85.
44. Bai J, Xie X, Lei Y, An G, He L, Lv X. Ocular albinism type 1-induced melanoma cell migration is mediated through the RAS/RAF/MEK/ERK signaling pathway. Mol Med Rep. 2014;10(1):491-5.
45. Gao Y, Zhao H, Ren M, Chen Q, Li J, Li Z, et al. TOP2A Promotes Tumorigenesis of High-grade Serous Ovarian Cancer by Regulating the TGF-beta/Smad Pathway. J Cancer. 2020;11(14):4181-92.
46. Jain M, Zhang L, He M, Zhang YQ, Shen M, Kebebew E. TOP2A is overexpressed and is a therapeutic target for adrenocortical carcinoma. Endocr Relat Cancer. 2013;20(3):361-70.
47. Park JS, Kim HS, Park MY, Kim CH, Chung YJ, Hong YK, et al. Topoisomerase II alpha as a universal tumor antigen: antitumor immunity in murine tumor models and H-2K(b)-restricted T cell epitope. Cancer Immunol Immunother. 2010;59(5):747-57.
48. Bhan S, Chuang A, Negi SS, Glazer CA, Califano JA. MAGEA4 induces growth in normal oral keratinocytes by inhibiting growth arrest and apoptosis. Oncol Rep. 2012;28(4):1498-502.
49. Daudi S, Eng KH, Mhawech-Fauceglia P, Morrison C, Miliotto A, Beck A, et al. Expression and immune responses to MAGE antigens predict survival in epithelial ovarian cancer. PLoS One. 2014;9(8):e104099.
50. Garcia-Soto AE, Schreiber T, Strbo N, Ganjei-Azar P, Miao F, Koru-Sengul T, et al. Cancer-testis antigen expression is shared between epithelial ovarian cancer tumors. Gynecol Oncol. 2017;145(3):413-9.
51. Kageyama S, Ikeda H, Miyahara Y, Imai N, Ishihara M, Saito K, et al. Adoptive Transfer of MAGE-A4 T-cell Receptor Gene-Transduced Lymphocytes in Patients with Recurrent Esophageal Cancer. Clin Cancer Res. 2015;21(10):2268-77.
52. Sanderson JP, Crowley DJ, Wiedermann GE, Quinn LL, Crossland KL, Tunbridge HM, et al. Preclinical evaluation of an affinity-enhanced MAGE-A4-specific T-cell receptor for adoptive T-cell therapy. Oncoimmunology. 2020;9(1):1682381.
53. Yakirevich E, Sabo E, Lavie O, Mazareb S, Spagnoli GC, Resnick MB. Expression of the MAGE-A4 and NY-ESO-1 cancer-testis antigens in serous ovarian neoplasms. Clin Cancer Res. 2003;9(17):6453-60.
54. Zimmermann AK, Imig J, Klar A, Renner C, Korol D, Fink D, et al. Expression of MAGE-C1/CT7 and selected cancer/testis antigens in ovarian borderline tumours and primary and recurrent ovarian carcinomas. Virchows Arch. 2013;462(5):565-74.
55. Berinstein NL, Karkada M, Oza AM, Odunsi K, Villella JA, Nemunaitis JJ, et al. Survivin-targeted immunotherapy drives robust polyfunctional T cell generation and differentiation in advanced ovarian cancer patients. Oncoimmunology. 2015;4(8):e1026529.
56. Gil-Kulik P, Krzyzanowski A, Dudzinska E, Karwat J, Chomik P, Swistowska M, et al. Potential Involvement of BIRC5 in Maintaining Pluripotency and Cell Differentiation of Human Stem Cells. Oxid Med Cell Longev. 2019;2019:8727925.
57. He X, Yang K, Wang H, Chen X, Wu H, Yao L, et al. Expression and clinical significance of survivin in ovarian cancer: A meta-analysis. PLoS One. 2018;13(5):e0194463.
58. Plewka D, Jakubiec-Bartnik B, Morek M, Bogunia E, Bienioszek M, Wolski H, et al. Survivin in ovary tumors. Ginekol Pol. 2015;86(7):525-30.
59. Yamamoto M, Suzuki S, Togashi K, Sanomachi T, Seino S, Kitanaka C, et al. AS602801, an Anticancer Stem Cell Candidate Drug, Reduces Survivin Expression and Sensitizes A2780 Ovarian Cancer Stem Cells to Carboplatin and Paclitaxel. Anticancer Res. 2018;38(12):6699-706.
60. Zhao G, Wang Q, Wu Z, Tian X, Yan H, Wang B, et al. Ovarian Primary and Metastatic Tumors Suppressed by Survivin Knockout or a Novel Survivin Inhibitor. Mol Cancer Ther. 2019;18(12):2233-45.
61. Brunette LL, Mhawech-Fauceglia PY, Ji L, Skeate JG, Brand HE, Lawrenson K, et al. Validity and prognostic significance of sperm protein 17 as a tumor biomarker for epithelial ovarian cancer: a retrospective study. BMC Cancer. 2018;18(1):970.
62. Chiriva-Internati M, Weidanz JA, Yu Y, Frezza EE, Jenkins MR, Kennedy RC, et al. Sperm protein 17 is a suitable target for adoptive T-cell-based immunotherapy in human ovarian cancer. J Immunother. 2008;31(8):693-703.

63. Gao Q, Xiang SD, Wilson K, Madondo M, Stephens AN, Plebanski M. Sperm Protein 17 Expression by Murine Epithelial Ovarian Cancer Cells and Its Impact on Tumor Progression. Cancers (Basel). 2018;10(8).
64. Kanduc D. Oligopeptides for Immunotherapy Approaches in Ovarian Cancer Treatment. Curr Drug Discov Technol. 2019;16(3):285-9.
65. Straughn JM, Jr., Shaw DR, Guerrero A, Bhoola SM, Racelis A, Wang Z, et al. Expression of sperm protein 17 (Sp17) in ovarian cancer. Int J Cancer. 2004;108(6):805-11.
66. Vermeij R, Daemen T, de Bock GH, de Graeff P, Leffers N, Lambeck A, et al. Potential target antigens for a universal vaccine in epithelial ovarian cancer. Clin Dev Immunol. 2010;2010.
67. Marijt KA, Blijleven L, Verdegaal EME, Kester MG, Kowalewski DJ, Rammensee HG, et al. Identification of non-mutated neoantigens presented by TAP-deficient tumors. J Exp Med. 2018;215(9):2325-37.
68. Odunsi K, Jungbluth AA, Stockert E, Qian F, Gnjatic S, Tammela J, et al. NY-ESO-1 and LAGE-1 cancer-testis antigens are potential targets for immunotherapy in epithelial ovarian cancer. Cancer Res. 2003;63(18):6076-83.
69. McCormack E, Adams KJ, Hassan NJ, Kotian A, Lissin NM, Sami M, et al. Bi-specific TCR-anti CD3 redirected T-cell targeting of NY-ESO-1- and LAGE-1-positive tumors. Cancer Immunol Immunother. 2013;62(4):773-85.
70. Rapoport AP, Stadtmauer EA, Binder-Scholl GK, Goloubeva O, Vogl DT, Lacey SF, et al. NY-ESO-1-specific TCR-engineered T cells mediate sustained antigen-specific antitumor effects in myeloma. Nat Med. 2015;21(8):914-21.
71. Cheng W, Huang PC, Chao HM, Jeng YM, Hsu HC, Pan HW, et al. Glypican-3 induces oncogenicity by preventing IGF-1R degradation, a process that can be blocked by Grb10. Oncotarget. 2017;8(46):80429-42.
72. Liu Y, Zheng D, Liu M, Bai J, Zhou X, Gong B, et al. Downregulation of glypican-3 expression increases migration, invasion, and tumorigenicity of human ovarian cancer cells. Tumour Biol. 2015;36(10):7997-8006.
73. Pantanowitz L, Otis CN. Glypican-3 immunohistochemistry in the ovary. Histopathology. 2008;53(1):115-7.
74. Shimizu Y, Suzuki T, Yoshikawa T, Endo I, Nakatsura T. Next-Generation Cancer Immunotherapy Targeting Glypican-3. Front Oncol. 2019;9:248.
75. Godelaine D, Carrasco J, Brasseur F, Neyns B, Thielemans K, Boon T, et al. A new tumor-specific antigen encoded by MAGE-C2 and presented to cytolytic T lymphocytes by HLA-B44. Cancer Immunol Immunother. 2007;56(6):753-9.
76. Lamers CH, van Steenbergen-Langeveld S, van Brakel M, Groot-van Ruijven CM, van Elzakker PM, van Krimpen B, et al. T cell receptor-engineered T cells to treat solid tumors: T cell processing toward optimal T cell fitness. Hum Gene Ther Methods. 2014;25(6):345-57.
77. Ma W, Germeau C, Vigneron N, Maernoudt AS, Morel S, Boon T, et al. Two new tumor-specific antigenic peptides encoded by gene MAGE-C2 and presented to cytolytic T lymphocytes by HLA-A2. Int J Cancer. 2004;109(5):698-702.
78. Song X, Hao J, Wang J, Guo C, Wang Y, He Q, et al. The cancer/testis antigen MAGEC2 promotes amoeboid invasion of tumor cells by enhancing STAT3 signaling. Oncogene. 2017;36(11):1476-86.
79. Straetemans T, van Brakel M, van Steenbergen S, Broertjes M, Drexhage J, Hegmans J, et al. TCR gene transfer: MAGE-C2/HLA-A2 and MAGE-A3/HLA-DP4 epitopes as melanoma-specific immune targets. Clin Dev Immunol. 2012;2012:586314.
80. Di Modugno F, Caprara V, Chellini L, Tocci P, Spadaro F, Ferrandina G, et al. hMENA is a key regulator in endothelin-1/beta-arrestin1-induced invadopodial function and metastatic process. Proc Natl Acad Sci U S A. 2018;115(12):3132-7.
81. Brennan DJ, Ek S, Doyle E, Drew T, Foley M, Flannelly G, et al. The transcription factor Sox11 is a prognostic factor for improved recurrence-free survival in epithelial ovarian cancer. Eur J Cancer. 2009;45(8):1510-7.
82. Choi MK, Seong I, Kang SA, Kim J. Down-regulation of Sox11 is required for efficient osteogenic differentiation of adipose-derived stem cells. Mol Cells. 2014;37(4):337-44.
83. Davidson B, Holth A, Hellesylt E, Tan TZ, Huang RY, Trope C, et al. The clinical role of epithelial-mesenchymal transition and stem cell markers in advanced-stage ovarian serous carcinoma effusions. Hum Pathol. 2015;46(1):1-8.
84. Fang G, Liu J, Wang Q, Huang X, Yang R, Pang Y, et al. MicroRNA-223-3p Regulates Ovarian Cancer Cell Proliferation and Invasion by Targeting SOX11 Expression. Int J Mol Sci. 2017;18(6).
85. Schmitz M, Wehner R, Stevanovic S, Kiessling A, Rieger MA, Temme A, et al. Identification of a naturally processed T cell epitope derived from the glioma-associated protein SOX11. Cancer Lett. 2007;245(1-2):331-6.
86. Sernbo S, Gustavsson E, Brennan DJ, Gallagher WM, Rexhepaj E, Rydnert F, et al. The tumour suppressor SOX11 is associated with improved survival among high grade epithelial ovarian cancers and is regulated by reversible promoter methylation. BMC Cancer. 2011;11:405.
87. Hayashi S, Kumai T, Matsuda Y, Aoki N, Sato K, Kimura S, et al. Six-transmembrane epithelial antigen of the prostate and enhancer of zeste homolog 2 as immunotherapeutic targets for lung cancer. J Transl Med. 2011;9:191.
88. Jiao Z, Huang L, Sun J, Xie J, Wang T, Yin X, et al. Six-transmembrane epithelial antigen of the prostate 1 expression promotes ovarian cancer metastasis by aiding progression of epithelial-to-mesenchymal transition. Histochem Cell Biol. 2020.
89. Moreaux J, Kassambara A, Hose D, Klein B. STEAP1 is overexpressed in cancers: a promising therapeutic target. Biochem Biophys Res Commun. 2012;429(3-4):148-55.
90. Vaghjiani RJ, Talma S, Murphy CL. Six-transmembrane epithelial antigen of the prostate (STEAP1 and STEAP2)-differentially expressed by murine and human mesenchymal stem cells. Tissue Eng Part A. 2009;15(8):2073-83.
91. Chen YT, Hsu M, Lee P, Shin SJ, Mhawech-Fauceglia P, Odunsi K, et al. Cancer/testis antigen CT45: analysis of mRNA and protein expression in human cancer. Int J Cancer. 2009;124(12):2893-8.
92. Coscia F, Lengyel E, Duraiswamy J, Ashcroft B, Bassani-Sternberg M, Wierer M, et al. Multi-level Proteomics Identifies CT45 as a Chemosensitivity Mediator and Immunotherapy Target in Ovarian Cancer. Cell. 2018;175(1):159-70 e16.

93. Shang B, Gao A, Pan Y, Zhang G, Tu J, Zhou Y, et al. CT45A1 acts as a new proto-oncogene to trigger tumorigenesis and cancer metastasis. Cell Death Dis. 2014;5:e1285.
94. Zhang W, Barger CJ, Link PA, Mhawech-Fauceglia P, Miller A, Akers SN, et al. DNA hypomethylation-mediated activation of Cancer/Testis Antigen 45 (CT45) genes is associated with disease progression and reduced survival in epithelial ovarian cancer. Epigenetics. 2015;10(8):736-48.
95. Llaurado M, Abal M, Castellvi J, Cabrera S, Gil-Moreno A, Perez-Benavente A, et al. ETV5 transcription factor is overexpressed in ovarian cancer and regulates cell adhesion in ovarian cancer cells. Int J Cancer. 2012;130(7):1532-43.
96. Chiste M, Alexis J, Recine M. IMP3 expression in serous tumors of the ovary. Appl Immunohistochem Mol Morphol. 2014;22(9):658-62.
97. El-Balat A, Sanger N, Karn T, Becker S, Holtrich U, Muallem Z, et al. IMP3 Expression in Borderline Tumors of the Ovary. Anticancer Res. 2017;37(2):583-8.
98. Hsu KF, Shen MR, Huang YF, Cheng YM, Lin SH, Chow NH, et al. Overexpression of the RNA-binding proteins Lin28B and IGF2BP3 (IMP3) is associated with chemoresistance and poor disease outcome in ovarian cancer. Br J Cancer. 2015;113(3):414-24.
99. Kobel M, Xu H, Bourne PA, Spaulding BO, Shih Ie M, Mao TL, et al. IGF2BP3 (IMP3) expression is a marker of unfavorable prognosis in ovarian carcinoma of clear cell subtype. Mod Pathol. 2009;22(3):469-75.
100. Lu L, Wang S, Zhu Q, Qu Y, Gu W, Ning Y, et al. The expression of IMP3 in 366 cases with ovarian carcinoma of high grade serous, endometrioid and clear cell subtypes. Pathol Res Pract. 2018;214(8):1087-94.
101. Tomita Y, Harao M, Senju S, Imai K, Hirata S, Irie A, et al. Peptides derived from human insulin-like growth factor-II mRNA binding protein 3 can induce human leukocyte antigen-A2-restricted cytotoxic T lymphocytes reactive to cancer cells. Cancer Sci. 2011;102(1):71-8.
102. Khalaf WS, Garg M, Mohamed YS, Stover CM, Browning MJ. In vitro Generation of Cytotoxic T Cells With Potential for Adoptive Tumor Immunotherapy of Multiple Myeloma. Front Immunol. 2019;10:1792.
103. Bekos C, Muqaku B, Dekan S, Horvat R, Polterauer S, Gerner C, et al. NECTIN4 (PVRL4) as Putative Therapeutic Target for a Specific Subtype of High Grade Serous Ovarian Cancer-An Integrative Multi-Omics Approach. Cancers (Basel). 2019;11(5).
104. Boylan KL, Buchanan PC, Manion RD, Shukla DM, Braumberger K, Bruggemeyer C, et al. The expression of Nectin-4 on the surface of ovarian cancer cells alters their ability to adhere, migrate, aggregate, and proliferate. Oncotarget. 2017;8(6):9717-38.
105. Derycke MS, Pambuccian SE, Gilks CB, Kalloger SE, Ghidouche A, Lopez M, et al. Nectin 4 overexpression in ovarian cancer tissues and serum: potential role as a serum biomarker. Am J Clin Pathol. 2010;134(5):835-45.
106. Lopez M, Ghidouche A, Rochas C, Godelaine D, Carrasco J, Colau D, et al. Identification of a naturally processed HLA-A*02:01-restricted CTL epitope from the human tumor-associated antigen Nectin-4. Cancer Immunol Immunother. 2016;65(10):1177-88.
107. Sethy C, Goutam K, Nayak D, Pradhan R, Molla S, Chatterjee S, et al. Clinical significance of a pvrl 4 encoded gene Nectin-4 in metastasis and angiogenesis for tumor relapse. J Cancer Res Clin Oncol. 2020;146(1):245-59.
108. AlHossiny M, Luo L, Frazier WR, Steiner N, Gusev Y, Kallakury B, et al. Ly6E/K Signaling to TGFbeta Promotes Breast Cancer Progression, Immune Escape, and Drug Resistance. Cancer Res. 2016;76(11):3376-86.
109. Benti S, Tiwari PB, Goodlett DW, Daneshian L, Kern GB, Smith MD, et al. Small Molecule Binds with Lymphocyte Antigen 6K to Induce Cancer Cell Death. Cancers (Basel). 2020;12(2).
110. Luo L, McGarvey P, Madhavan S, Kumar R, Gusev Y, Upadhyay G. Distinct lymphocyte antigens 6 (Ly6) family members Ly6D, Ly6E, Ly6K and Ly6H drive tumorigenesis and clinical outcome. Oncotarget. 2016;7(10):11165-93.
111. Sastry NG, Wan X, Huang T, Alvarez AA, Pangeni RP, Song X, et al. LY6K Promotes Glioblastoma Tumorigenicity via CAV-1-Mediated ERK1/2 Signaling Enhancement. Neuro Oncol. 2020.
112. Tomita Y, Yuno A, Tsukamoto H, Senju S, Kuroda Y, Hirayama M, et al. Identification of immunogenic LY6K long peptide encompassing both CD4(+) and CD8(+) T-cell epitopes and eliciting CD4(+) T-cell immunity in patients with malignant disease. Oncoimmunology. 2014;3:e28100.
113. Sinnathamby G, Zerfass J, Hafner J, Block P, Nickens Z, Hobeika A, et al. ADAM metallopeptidase domain 17 (ADAM17) is naturally processed through major histocompatibility complex (MHC) class I molecules and is a potential immunotherapeutic target in breast, ovarian and prostate cancers. Clin Exp Immunol. 2011;163(3):324-32.
114. Gillespie AM, Rodgers S, Wilson AP, Tidy J, Rees RC, Coleman RE, et al. MAGE, BAGE and GAGE: tumour antigen expression in benign and malignant ovarian tissue. Br J Cancer. 1998;78(6):816-21.
115. Kerkar SP, Wang ZF, Lasota J, Park T, Patel K, Groh E, et al. MAGE-A is More Highly Expressed Than NY-ESO-1 in a Systematic Immunohistochemical Analysis of 3668 Cases. J Immunother. 2016;39(4):181-7.
116. Zhang S, Zhou X, Yu H, Yu Y. Expression of tumor-specific antigen MAGE, GAGE and BAGE in ovarian cancer tissues and cell lines. BMC Cancer. 2010;10:163.
117. Monji M, Nakatsura T, Senju S, Yoshitake Y, Sawatsubashi M, Shinohara M, et al. Identification of a novel human cancer/testis antigen, KM-HN-1, recognized by cellular and humoral immune responses. Clin Cancer Res. 2004;10(18 Pt 1):6047-57.
