# Supplementary material for: Target Selection for T-Cell Therapy in Epithelial Ovarian Cancer: Systematic Prioritization of Self-Antigens
Source: Int J Mol Sci. 2023 Jan 24;24(3):2292. doi: 10.3390/ijms24032292 (PMC9916968; doi:10.3390/ijms24032292)
Supplement: Supplementary file 1 [file ijms-24-02292-s001.zip › Supplemental Table S4 IEDB Analysis of Peptides.pdf]

Supplemental Table S4 – predescribed Peptides for TAAs by mass spectrometry

Antigen	Epitopes identified	Epitopes identified on Cancer Cells	Epitopes identified in Ovarian Cancer	HLA-A*2:01 restricted epitopes*
KIF20A	133	75	4	16
CT45	9	7	0	2
LY6K	1	1	1	/
MAGEA4	36	35	0	7
PRAME	97	75	2	12
SP17	12	7	0	0

* number of HLA-A*2:01 restricted epitopes out of total epitopes identified
