# Supplementary material for: Target Selection for T-Cell Therapy in Epithelial Ovarian Cancer: Systematic Prioritization of Self-Antigens
Source: Int J Mol Sci. 2023 Jan 24;24(3):2292. doi: 10.3390/ijms24032292 (PMC9916968; doi:10.3390/ijms24032292)
Supplement: Supplementary file 1 [file ijms-24-02292-s001.zip › Supplemental Table S5.pdf]

Supplemental Table S5

Results of explorative data analysis

Analysis of contingency tables. Table b) shows p-values calculated with Fisher's exact test

Table a) Contingency Table p-values (2-sided significance) Fisher's exact test; brackets show the groups that were compared to each other

	Tumor Grade	FIGO	Local vs. progressed (T-Stage T1/T2 vs. T3)	TNM T-Stage	TNM N-Stage	TNM M-Stage	Peritoneal carcinomatosis	Platinum Response	PDL1 Intensity
**KIF20A Intensity** (no vs. weak vs. moderate vs. strong staining)	0,225	0,932	0,727	0,366	0,574	0,986	0,335	0,607	0,393
**KIF20A_Int1** (no, weak and moderate staining vs. strong staining)	0,189	0,863	1	0,473	0,56	0,929	0,664	0,558	0,365
**KIF20A_Int 2** (no and weak staining vs. moderate and strong staining)	1	0,654	0,324	0,314	0,294	0,802	0,324	0,467	0,289
**KIF20A_Q1** (Q-Score 0-100 vs. Q-Score 101-300)	1	0,654	0,324	0,314	0,294	0,802	0,324	0,467	0,289
**KIF20A_Q2** (Q-Score 0-200 vs. Q-Score 201-300)	0,175	0,747	1	0,526	0,311	0,859	0,656	0,766	0,592
**KIF20A_Q3** (Q-Score 0 vs. Q-Score 1-200 vs. Q-Score 201-300)	0,189	0,863	1	0,473	0,56	0,929	0,664	0,558	0,365
**KIF20A_Q4** (Q-Score 0 vs. Q-Score 1-149 vs. Q-Score 150-300)	1	0,654	0,324	0,314	0,294	0,802	0,324	0,467	0,289
**KIF20A_Q5** (Q-Score < 150 vs. Q-Score ≥ 150)	1	0,654	0,324	0,314	0,294	0,802	0,324	0,467	0,289
**CT45 Intensity** (no vs. weak vs. moderate vs. strong staining)	1	0,425	0,029	0,255	0,984	0,542	0,136	0,293	0,682
**CT45_Int1** (no, weak and moderate staining vs. strong staining)	1	0,092	0,009	0,025	1	1	0,046	0,462	0,897
**CT45_Int2** (no and weak staining vs. moderate and strong staining)	1	0,436	0,212	0,242	1	0,266	0,397	0,378	0,818
**CT45_Q1** (Q-Score 0-100 vs. Q-Score 101-300)	1	0,34	0,18	0,148	1	0,628	0,415	0,242	0,75
**CT45_Q2** (Q-Score 0-200 vs. Q-Score 201-300)	0,552	0,068	0,005	0,018	1	0,873	0,052	0,7	0,795
**CT45_Q3** (Q-Score 0 vs. Q-Score 1-200 vs. Q-Score 201-300)	1	0,16	0,005	0,045	0,94	0,68	0,038	0,848	0,428
**CT45_Q4** (Q-Score 0 vs. Q-Score 1-149 vs. Q-Score 150-300)	0,841	0,362	0,105	0,406	0,825	0,211	0,485	0,039	0,739
**CT45_Q5** (Q-Score < 150 vs. Q-Score ≥ 150)	0,629	0,269	0,054	0,103	0,568	0,917	0,346	0,063	1

	Tumor Grade	FIGO	Local vs. progressed (T-Stage T1/T2 vs. T3)	TNM T-Stage	TNM N-Stage	TNM M-Stage	Peritoneal carcinomatosis	Platinum Response	PDL1 Intensity
**LY6K Intensity** (no vs. weak vs. moderate vs. strong staining)	0,409	0,637	0,301	0,582	0,022	0,399	1	0,228	0,903
**LY6K_Int1** (no, weak and moderate staining vs. strong staining)	1	1	1	0,228	0,333	1	1	1	1
**LY6K_Int2** (no and weak staining vs. moderate and strong staining)	0,353	0,173	0,105	0,791	0,03	0,343	1	0,099	1
**LY6K_Q1** (Q-Score 0-100 vs. Q-Score 101-300)	0,192	1	0,095	0,292	0,112	0,75	1	0,777	0,219
**LY6K_Q2** (Q-Score 0-200 vs. Q-Score 201-300)	1	1	1	0,228	0,333	1	1	1	1
**LY6K_Q3** (Q-Score 0 vs. Q-Score 1-200 vs. Q-Score 201-300)	1	0,747	1	0,469	0,063	0,602	1	0,770	0,7
**LY6K_Q4** (Q-Score 0 vs. Q-Score 1-149 vs. Q-Score 150-300)	0,231	0,569	0,116	0,915	0,098	0,529	0,379	0,643	0,408
**LY6K_Q5** (Q-Score < 150 vs. Q-Score ≥ 150)	0,17	0,444	0,006	0,18	0,151	0,548	0,226	0,571	0,31

Table b) Spearman's-Rho correlation KIF20A, CT45, LY6K Q-Score (metric) and clinical parameters

Spearman's rho		KIF20A_Qscore	CT45_Qscore	LY6K_Qscore
**FIGO Classification**	Correlation Coefficient	,117	-,197	-,068
	Sig. (2-tailed)	,408	,141	,617
**Tumor Grade**	Correlation Coefficient	-,164	,008	-,232
	Sig. (2-tailed)	,244	,953	,082
**local vs. progressed**	Correlation Coefficient	,086	-,373	-,318
	Sig. (2-tailed)	,542	,004	,016
**TNM-Classification T**	Correlation Coefficient	,182	-,065	-,269
	Sig. (2-tailed)	,196	,631	,043
	N	52	57	57
**TNM-Classification N**	Correlation Coefficient	,099	-,004	,147
	Sig. (2-tailed)	,485	,979	,276
**TNM-Classification M**	Correlation Coefficient	,060	,109	,064
	Sig. (2-tailed)	,677	,423	,640
**peritoneal_ carcinomatosis**	Correlation Coefficient	,004	,124	,153
	Sig. (2-tailed)	,976	,358	,257
**Platinumresponse after 1st Line Chemotherapy**	Correlation Coefficient	,141	,093	,117
	Sig. (2-tailed)	,320	,496	,392
